# Estimating decades-long trends in petroleum field energy return on investment (EROI) with an engineering-based model

**DOI:** 10.1371/journal.pone.0171083

**Published:** 2017-02-08

**Authors:** Vinay S. Tripathi, Adam R. Brandt

**Affiliations:** Department of Energy Resources Engineering, Stanford University, Stanford, California, United States of America; Centro de Investigacion Cientifica y de Educacion Superior de Ensenada Division de Fisica Aplicada, MEXICO

## Abstract

This paper estimates changes in the energy return on investment (EROI) for five large petroleum fields over time using the Oil Production Greenhouse Gas Emissions Estimator (OPGEE). The modeled fields include Cantarell (Mexico), Forties (U.K.), Midway-Sunset (U.S.), Prudhoe Bay (U.S.), and Wilmington (U.S.). Data on field properties and production/processing parameters were obtained from a combination of government and technical literature sources. Key areas of uncertainty include details of the oil and gas surface processing schemes. We aim to explore how long-term trends in depletion at major petroleum fields change the effective energetic productivity of petroleum extraction. Four EROI ratios are estimated for each field as follows: The net energy ratio (NER) and external energy ratio (EER) are calculated, each using two measures of energy outputs, (1) oil-only and (2) all energy outputs. In all cases, engineering estimates of inputs are used rather than expenditure-based estimates (including off-site indirect energy use and embodied energy). All fields display significant declines in NER over the modeling period driven by a combination of (1) reduced petroleum production and (2) increased energy expenditures on recovery methods such as the injection of water, steam, or gas. The fields studied had NER reductions ranging from 46% to 88% over the modeling periods (accounting for all energy outputs). The reasons for declines in EROI differ by field. Midway-Sunset experienced a 5-fold increase in steam injected per barrel of oil produced. In contrast, Prudhoe Bay has experienced nearly a 30-fold increase in amount of gas processed and reinjected per unit of oil produced. In contrast, EER estimates are subject to greater variability and uncertainty due to the relatively small magnitude of external energy investments in most cases.

## Introduction

This paper is adapted from the M.S. thesis of Tripathi for publication in PLOS ONE [1].

### Energy return on investment

Monetary flows shape the behavior of individuals and countries. This behavior includes the evaluation of energy resources, which are typically judged using the measures of monetary returns. However, monetary accounting has been criticized for providing an incomplete assessment of energy resource quality. The measurement of energy flows associated with an energy resource was posed as an alternate quality assessment framework by Odum [2]. Odum argued that energetic metrics offer a more accurate, physics-based evaluation of a primary energy resource’s true utility [2]. Within this framework, Hall et al. defined energy return on investment (EROI) as the ratio of energy production to the required energy inputs associated with producing a primary energy resource [3]. EROI has been estimated using a variety of methods and definitions for many types of energy resources, including petroleum fields.

Murphy, et al. provide a method for defining the EROI boundary consisting of two variables: (1) the boundary at which energetic returns are measured, and (2) the boundary at which energetic investments are estimated [4]. Their method includes a proposed “standard” EROI and their paper summarizes the details of EROI estimation [4]. In this typology, ratios with boundary “1” include only extraction of energy sources, while ratios with boundary “2” also include refining or processing. Murphy et al. also classify EROIs by inclusion of only direct inputs “d”, or including both direct and indirect inputs “i”. EROI_*1,i*_ serves as the standard EROI within the Murphy et al. system [4].

Several recent studies have estimated the EROI of various petroleum resources over time. An example is the analysis of the Canadian petroleum industry by Poisson and Hall [5]. They use data from the Canadian government on the direct energy consumption of the Canadian petroleum sector to estimate the energy investment used in calculating EROI_*1,d*_ [5]. They estimate the Canadian petroleum sector’s combined direct and indirect energy consumption as the product of the sector’s energy intensity factor [units energy/units currency] and the financial value of the sector’s hydrocarbon production. They estimate that Canadian petroleum production EROI_*stnd*_ declined by 13% during the 1990-2008 period [5].

Another temporal EROI analysis focuses on the Russian petroleum sector [6]. Nogovitsyn and Sokolov use direct energy consumption reports to estimate EROI for the overall Russian petroleum industry and for two major Russian natural gas producing companies, Gazprom and Novatek [6]. Nogovitsyn and Sokolov estimate that the NER_*dev. and transp.*_ (similar to EROI_*1,d*_) of the overall Russian petroleum sector decreased by 17% during the 2005-2012 period [6].

Hu et al. estimate several EROI ratios for China’s Daqing field, including EROI_*1,d*_ and EROI_*stnd*_, using energy and financial expenditures flowing into Daqing and Chinese industrial energy intensity factors [7]. During 2001-2009 they estimate that Daqing’s EROI_*1,d*_ declined by 22% and its EROI_*stnd*_ declined by 35%. Daqing’s EROI decline profiles were fairly smooth over the 2001-2009 period [7].

In another recent work, a model based on engineering principles is used to estimate a current EROI for forty petroleum fields [8]. Brandt et al. obtain data on field properties and extraction practices. The engineering-based model then estimates the energy investments required to perform these petroleum field operations. Brandt et al. estimate two types of EROI: a net energy return (NER) and an external energy return (EER). While this NER is noted as comparable to EROI_*stnd*_, their model did not include embodied material inputs. Brandt et al. found great variation in the estimated EROI for the various fields; factors such as higher intensity of enhanced oil recovery operations resulted in fields with relatively lower EROIs [8]. An earlier temporal analysis of onshore oil fields in California, U.S. used a simpler model also based on engineering principles [9]. Brandt estimates that during the 1955-2005 period the NER of California oil fields declined by approximately 92% (starting at 63 and ending at 5, approximately) [9]. When crude refining is added, NER declined by only 44% during 1955-2005 due to a lower initial EROI value.

Temporal EROI analysis has also been applied to unconventional hydrocarbon resources [10]. Brandt, Englander et al. analyze the direct energy consumption input and output flows of the Alberta, Canada bitumen industry to estimate several NER and EER ratios during 1970-2010 [10]. Their “mine mouth” and “point of use” NERs are similar to EROI_*1,d*_ and EROI_*2,d*_, respectively, of Murphy et al. but do not include embodied energy inputs [4, 10]. They estimate that mine-mouth NER values of Alberta bitumen production were generally stable during 1970-2010, remaining around 5. Notably, their estimated “mine mouth” EER values for bitumen produced using mining methods are significantly higher and more variable because processed bitumen is used to power a significant portion of oil sands.

The methodology of this work is similar to that of Brandt et al [8], but here we shift the focus toward a deep temporal analysis of a relatively small (but diverse) set of very large petroleum fields over decades. The temporal field-level focus of this paper is similar to the scope of Hu et al. [7]. Additionally, this analysis considers indirect consumption of energy embodied in the manufacturing of petroleum field materials, wells, and equipment.

## Materials and methods

### Introduction to methods

Five petroleum fields were selected for analysis: Wilmington and Midway-Sunset in the U.S. (California), Cantarell in Mexico, Forties in the U.K., and Prudhoe Bay in the U.S. (Alaska). The objective is to track the EROI of large fields over a long period of time: a quarter-century or longer, if possible. It is expected that estimated EROI values will decline for all petroleum fields, but the precise decline profiles are unknown.

All of the selected fields are “giants” with at least 2.9 billion barrels of estimated ultimately recoverable reserves (URR). This study focuses on giant fields because, while relatively few in number, they account for a large share of global petroleum production [11]. The fields selected represent a range of reservoir parameters and production practices. The fields include onshore and offshore fields and fields with heavy and light oils. The reservoirs vary with regard to key factors such as depth and water-oil-ratio. These reservoir parameters in turn affect post-primary recovery production practices.

### Introduction to the oil production greenhouse gas emissions estimator (OPGEE) model

The EROI of each petroleum field is estimated over time using the Oil Production Greenhouse Gas Emissions Estimator (OPGEE) [12]. OPGEE v2.0a was used with minor modifications to drilling energy estimates. OPGEE calculates greenhouse gas emissions based on the energy consumption of a petroleum field’s production operations [13]. OPGEE can therefore be used to model the energy invested into a field. Energy contained within the oil and within any exports of natural gas, natural gas liquids (NGLs), and electricity is the basis for calculating the energy returns from a petroleum field.

OPGEE receives parameters regarding the functioning of a petroleum field. These include the choice of production processes such gas lift, basic reservoir parameters such as average pressure, and choices regarding the processing of crude oil and natural gas. When input data is not available, OPGEE applies or calculates default values based on the literature [14].

OPGEE then uses engineering principles and technical data to estimate the energy requirements of the major production steps. These calculations are divided into processing stages. The stages considered in this analysis are *Embodied Emissions*, *Drilling*, *Production & Extraction*, *Surface Processing*, and *Crude Transport*. Table 1 summarizes the energy sources used in the processes associated with each OPGEE upstream stage. El-Houjeiri, Brandt, and Duffy provide an overview of OPGEE’s structure and example calculations [13]. Brandt documents OPGEE’s estimation of the energy embodied in petroleum field equipment, facilities, and materials [15].

**Table 1:** The energy sources used in the processes associated with each OPGEE upstream stage. Data from Brandt et al. [8].

OPGEE Stage	Energy Source	OPGEE Process
Drilling & Development	Diesel	Drilling
Production & Extraction	Electricity^a^	N_2_ air separation unit
	Natural gas	Downhole pump, water re-injection pump, natural gas re-injection compressor, water flooding injection pump, gas lifting compressor, gas flooding injection compressor, steam generation
Surface Processing	Electricity^a^	Amine treater (pumps and air coolers), glycol dehydrator (glycol pump), water treatment
	Natural gas	Heater/treater, stabilizer column, amine treater (reboiler), demethanizer, glycol dehydrator (reboiler)
Transport^b^	Diesel or fuel oil	Barges, railroads, tankers
	Electricity^a^	Pipelines
Embodied Emissions	Various	All

^a^ Electricity for Cantarell, Prudhoe Bay, and Forties is produced on-site. Electricity at Wilmington and Midway-Sunset is imported from the grid.

^b^ Oil for all fields is transported to Houston, U.S. to be refined to provide a constant basis of comparison of transport-related energy investments.

### Using OPGEE to estimate EROI

**Fig 1:**
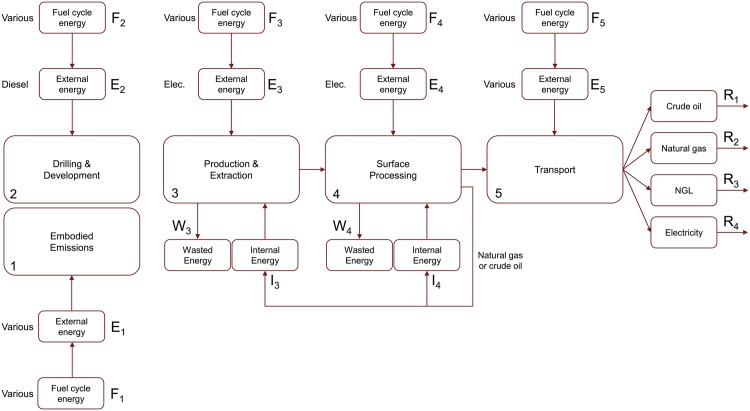
Schematic of OPGEE production processes and related energy flows used to calculate NER and EER values. Figure adapted from Brandt et al. [8].

Estimation of EROI is performed by calculating the net energy ratio (NER) and external energy ratio (EER) for each field according to the general procedure from Brandt et al. [8]. Fig 1 is a schematic of the OPGEE processing stages used in this analysis and accompanying energy investments, energy returns, and energy waste flows, adapted from Brandt et al. [8]. Subscripts 1-5 correspond to the OPGEE process stage associated with a given flow.

The energy flows are now defined, for process stage *n* and time-step *t* as:

1. Fuel Cycle Energy Investments, *F*_*n,t*_
2. External Energy Investments, *E*_*n,t*_
3. Internal Energy Investments, *I*_*n,t*_

External energy investments *E*_*n,t*_ represent energy originating from outside the petroleum field imported to run operations. An example is electricity imported for water treatment processes. Fuel cycle energy investments *F*_*n,t*_ represent the energy consumed to produce external energy investments. Internal energy investments *I*_*n,t*_ consist of energy produced at the petroleum field that is used within the petroleum field, rather than being exported. An example is combustion of natural gas to operate acid gas removal (AGR) units.

There are two categories of energy outflows proceeding from the processing stages:

1. Wasted Energy Flows, *W*_*n,t*_
2. Energy Returns, *R*_*n,t*_

Wasted energy flows represent loss of associated gas from operational flaring, venting, or fugitive emissions. It is assumed here that waste energy flows emanate only from the *Production & Extraction* and *Surface Processing* stages. Energy returns represent the energy content of the hydrocarbons (oil, natural gas, or NGLs) leaving the system as well as exported electricity generated at the field.

Instead of being exported, a portion of the produced natural gas is routed for use as internal energy flows (*I*_*3*_ and *I*_*4*_) that drive processes within *Production & Extraction* and *Surface Processing*. In the case of the early Midway-Sunset and Wilmington modeling periods, *I*_*3*_ also contains crude oil combusted to generate steam. All five OPGEE process stages also receive external energy investments *E*_*n,t*_ (diesel, electricity or residual fuel).

Using these energy investment and returns flows, two types of EROI ratios are calculated: the net energy return (NER) and the external energy return (EER). Additionally, NER and EER are each calculated in two ways: (1) by including only the energy returns from oil (NER_*oil*_ and EER_*oil*_), and (2) by including all energy returns (NER_*total*_ and EER_*total*_). Thus, a total of four EROI ratios are calculated for each field. Following Brandt et al. the ratios are conceptually defined in Eqs (1)–(4) as [8]:

$$\begin{array}{c} NER_{oil,t}=\frac{R_{1,t}}{\sum_{n=1}^{5}F_{n,t}+\sum_{n=1}^{5}E_{n,t}+\sum_{n=1}^{5}I_{n,t}} \end{array}$$ (1)

$$\begin{array}{c} NER_{total,t}=\frac{\sum_{n=1}^{4}R_{n,t}}{\sum_{n=1}^{5}F_{n,t}+\sum_{n=1}^{5}E_{n,t}+\sum_{n=1}^{5}I_{n,t}} \end{array}$$ (2)

$$\begin{array}{c} EER_{oil,t}=\frac{R_{1,t}}{\sum_{n=1}^{5}F_{n,t}+\sum_{n=1}^{5}E_{n,t}} \end{array}$$ (3)

$$\begin{array}{c} EER_{total,t}=\frac{\sum_{n=1}^{4}R_{n,t}}{\sum_{n=1}^{5}F_{n,t}+\sum_{n=1}^{5}E_{n,t}} \end{array}$$ (4)

The system boundaries of the energy investments used to calculate NER are similar—but not equivalent—to those of EROI_*stnd*_ in the method of Murphy et al. [4]. This analysis considers the energy requirements of extraction at the five fields. It also considers the energy required to prepare and transport crude oil for refining, but the energy required to refine the oil is excluded. The energy requirements of processing and transportation of produced natural gas are also included. Because the EROI_*stnd*_ of Murphy et al. includes only the energy requirements of extraction, our system boundary is more inclusive along this dimension [4].

This analysis considers direct and indirect energy inputs. However, all embodied energy requirements, which are a component of indirect energy inputs, are not included. OPGEE does not include the energy costs of producing consumed materials such as chemicals used for natural gas processing or new metals used during well maintenance. It is also assumed that capital investment in processing equipment such as compressors occurs only once. Energy requirements for construction of the drilling machinery and other field structures are also not considered in OPGEE. Our system boundary is thus less inclusive along this dimension of the EROI_*stnd*_ of Murphy et al. [4].

Henceforth the term “modeling period” is used to denote the years during which each is field is analyzed. For example, 1974-1999 is the Forties field modeling period.

OPGEE’s *Energy Consumption* sheet contains summary calculations of energy flows [12]. Energy investment flows *F*_*n,t*_, *E*_*n,t*_, and *I*_*n,t*_ are aggregated within these summary tables. These summary tables and other OPGEE model cells are used to calculate NER and EER as follows: Table 2 contains references to the OPGEE sheet and cell numbers to calculate energy energy investment and returns flows, based partially on the method of Brandt et al. [8]. The following abbreviations are used for OPGEE sheet names: EC = *Energy Consumption*, FS = *Fuel Specs*, R = *Results*, AF = *Active Field*. For example, “AF J63” refers to cell J63 within the *Active Field* sheet.

**Table 2:** Calculation of NER and EER flows from OPGEE model cells. Calculations based partially on the method of Brandt et al. [8].

Energy Flow	Description	Calculation
*R*_*oil,t*_	Energy returns as oil	(AF J63) ⋅ (FS M14)
*R*_*gas,t*_	Energy returns as gas	If -(EC E107) > 0, then -(EC E107)—(EC E138). Else 0.
*R*_*ngl,t*_	Energy returns as NGL	If -(EC E108) > 0, then -(EC E108). Else 0.
*R*_*elec,t*_	Energy returns as electricity	If -(EC E111) > 0, then -(EC E111). Else 0.
*I*_*gross,t*_	Gross energy investments	SUM(EC E93: EC E100)
*I*_*ext,t*_	External energy investments	SUMIF(EC E107: EC E111, > 0) + SUMIF(EC E113: EC E114, > 0)^a^
*I*_*fc,t*_	Fuel cycle energy investments	SUMIF(EC E138: EC E141, > 0) + SUMIF(EC E143, > 0)^a^
*I*_*emb,t*_	Embodied energy investments	EC E150

^a^ Use Microsoft Excel SUMIF function to only include a cell value in the sum if the cell value is greater than zero (signifying imports).

The flows calculated in Table 2 are used to calculate NER and EER values in Eqs (5)–(8):

$$\begin{array}{c} NER_{oil,t}=\frac{R_{oil,t}}{I_{gross,t}+I_{fc,t}+I_{emb,t}} \end{array}$$ (5)

$$\begin{array}{c} NER_{total,t}=\frac{R_{oil,t}+R_{gas,t}+R_{ngl,t}+R_{elec,t}}{I_{gross,t}+I_{fc,t}+I_{emb,t}} \end{array}$$ (6)

$$\begin{array}{c} EER_{oil,t}=\frac{R_{oil,t}}{I_{ext,t}+I_{fc,t}+I_{emb,t}} \end{array}$$ (7)

$$\begin{array}{c} EER_{total,t}=\frac{R_{oil,t}+R_{gas,t}+R_{ngl,t}+R_{elec,t}}{I_{ext,t}+I_{fc,t}+I_{emb,t}} \end{array}$$ (8)

Note that energy returns from natural gas, *R*_*gas,t*_, are calculated slightly differently than energy returns from NGLs, *R*_*ngl,t*_, and energy returns from electricity, *R*_*elec,t*_. *R*_*gas,t*_ contains the energy content of both the exported natural gas and the fuel cycle costs that would have been associated with its production, had the gas been produced elsewhere from the modeled petroleum field. In contrast, *R*_*ngl,t*_ and *R*_*elec,t*_ contain only the energy content of the exported NGLs or electricity, respectively.

OPGEE’s treatment of the energy required for drilling was modified for this analysis. OPGEE’s standard treatment uses an “expected lifetime well productivity” factor [bbl oil/well drilled], based on analysis of approximately one century of drilling and oil production statistics in California. This factor is used to calculate a drilling energy intensity factor [MMBtu consumed during drilling/MMBtu oil produced] approximated over the lifetime of a field [14]. To focus instead on the energy consumption of drilling for a particular year—rather than energy consumption distributed over the life a field—OPGEE was modified to calculate the drilling energy intensity factor for each year including only the wells drilled in that year (see [1] for more details).

## Data collection and adaptation for use in OPGEE

Historical operating statistics and reservoir parameters were obtained for each field to allow the use of OPGEE to estimate its EROI. If necessary, temporal data were converted to daily average rates for each year that a field was modeled. An example is the Alaska Oil and Gas Conservation Commission’s reporting of total monthly water production at Prudhoe Bay [16]. For a given year the total monthly water production was summed to obtain a yearly total and then divided by 365 to obtain a daily rate for that particular year.

Data quality for each OPGEE input is assessed described using a qualitative three-star scale. A three-star rating indicates that actual data (from government statistics or peer-reviewed literature) were available covering nearly the entire modeling period. An example of a three-star data source is the production rate of associated gas for all five fields. Three-star-rated data are still subject to a degree of variability or uncertainty associated with uncommon errors such as mis-measures or mis-reports.

A two-star rating indicates that complete data were not available; partial data required extrapolations, approximations, or an approximate field-level estimate, if applicable. The API gravity for all five fields is an example of a two-star rated parameter.

A one-star rating is used when data were not available, or if availability was mostly incomplete. An example is the molecular composition of the associated gas produced at the Cantarell, Midway-Sunset, Wilmington, and Forties fields. Also, the specific processing steps used in separation of oil and produced water (e.g. stabilizer column) or the choice of associated gas treatment processes (e.g. dehydrator) were not typically available.

In some cases the software program WebPlotDigitizer was used to extract data from plots and graphs in the literature [17]. Because the precise number of data points used in the generation of line plots in the literature is not typically clear, the process of data extraction and conversion to a yearly rate was approximate. An example is the data on the amount of natural gas produced and reinjected at Cantarell from Lozada et al. [18]. If data were obtained from a scatterplot, points were selected along the visually estimated “center mass” of the plotted data.

Multiple types of stimulation and artificial lift have been applied to the fields in this analysis. An example is the application of downhole pumping and gas lifting at the Forties field. During certain years, some wells at Forties used downhole pumping, some gas lifting, and some were free-flowing. It is not possible within OPGEE to simultaneously model all types of production practices, so in these cases the overall EROI is estimated by computing results for each method separately and then weighting the results by importance of each method.

In the literature, lifting gas injection was typically described as a rate of gas injected per day at a given producer well [scf/d]. The OPGEE input parameter “gas lifting injection ratio” has units of [scf/bbl liquid]. We therefore use total liquid production (oil and water) and the number of total producer wells to estimate a field-level ratio per bbl. This ratio is a sensitivity analysis parameter for all fields modeled with gas lift (see the Sensitivity Analysis section).

For fields that reinject natural gas, OPGEE allows the user to specify the fraction of remaining natural gas injected (henceforth FRNGI). In some cases, if 100% of gas is set to be reinjected, OPGEE will import additional gas to run processing equipment. In these cases, OPGEE is used to iteratively adjust the proportion of remaining natural gas that is reinjected until imports are approximately zero. To obtain a closer convergence to zero imports, OPGEE’s default iteration step size was reduced in this analysis (see [1]).

Table 3 depicts the source (user-inputted vs OPGEE default) for parameters in OPGEE’s main “Inputs” sheet that are relevant to this analysis. A user-inputted parameter was based on literature or on modeling assumptions. Because of variation in the OPGEE production practices applied to each field, all parameters are not relevant to all fields. For example, neither water reinjection nor water flooding were applied at Cantarell during any years of the modeling period. Therefore, in Table 3 the water injection ratio parameter is not applicable (NA) to Cantarell. Some user-inputted production method parameter values are equivalent to OPGEE’s default values. For example, gas flooding is not applied at Wilmington because it was not indicated in the literature. By default, OPGEE does not apply gas flooding either. Because this parameter was determined from the literature, it is treated as a user-inputted parameter within Table 3.

**Table 3:** Data classification for parameters in OPGEE’s main “Inputs” sheet: User-inputted (U) vs OPGEE default (D) (C = Cantarell, F = Forties, MS = Midway-Sunset, PB = Prudhoe Bay, W = Wilmington). User-inputted parameters are based on literature or modeling assumptions.

Parameter Type	Parameter	C	F	MS	PB	W
Production Methods	Downhole Pump (Y/N)	U	U	U	U	U
	Water Reinjection (Y/N)	U	U	U	U	U
	Natural Gas Reinjection (Y/N)	U	U	U	U	U
	Water Flooding (Y/N)	U	U	U	U	U
	Gas Lifting (Y/N)	U	U	U	U	U
	Gas Flooding (Y/N)	U	U	U	U	U
	Steam Flooding (Y/N)	U	U	U	U	U
Field Properties	Field Depth	U	U	U	U	U
	Oil Production Volume	U	U	U	U	U
	Number of Producing Wells	U	U	U	U	U
	Number of Water Injecting Wells	D^a^	U	U	U	U
	Well Diameter	NA	U	D	U	D
	Productivity Index	NA	D	D	D	D
	Reservoir Pressure	U	U	U	U	U
	Offshore (Y/N)	U	U	U	U	U
	New Wells Drilled	U	U	U	U	U
Fluid Properties	API Gravity	U	U	U	U	U
	Gas Composition	D	D	D	U	D
Production Practices	Gas-Oil-Ratio	U	U	U	U	U
	Water-Oil-Ratio	U	U	U	U	U
	Water Injection Ratio	NA	U	U	U	U
	Gas Lifting Injection Ratio	U	U	NA	U	NA
	Gas Flooding Injection Ratio	U	NA	NA	NA	NA
	Flood Gas	U	NA	NA	NA	NA
	Steam-Oil Ratio	NA	NA	U	NA	U
	Fraction of Req. Electricity Generated Onsite	U	U	U	U	U
	Fraction of Remaining Gas that is Reinjected	U	NA	U	U	NA
	Fraction of Water Produced that is Reinjected	NA	U	U	U	U
	Fraction of Steam Generation via Cogeneration	NA	NA	U	NA	U
Processing Practices	Heater/Treater (Y/N)	D	D	D	U	D
	Stabilizer Column (Y/N)	D	D	D	U	D
	Associated Gas Processing Path	D	D	D	U	D
	Flaring-Oil Ratio	D	D	D	D	D
	Venting-Oil Ratio	D	D	D	D	D
	Fraction of Oil Transported by Each Mode	U	U	U	U	U
Crude Oil Transport	Transport Distance	U	U	U	U	U
	Ocean Tanker Size	D	D	D	D	D

^a^ While water reinjection/flooding is not applied to Cantarell, OPGEE uses its default number of water injection wells in the calculation of embodied energy requirements for all fields.

While water injection is not applied at Cantarell, the OPGEE default value for the number of water injecting wells is used to calculate embodied energy requirements for all fields. This use of the default water injecting wells value at Cantarell is reflected in Table 3.

Table 4 presents an abbreviated data quality assessment for some key OPGEE input parameters for the five fields. Table 5 contains the literature sources for these parameters. Tables containing detailed input parameters, quality assessments, sources, and modeling notes on the fields are available in Tripathi [1]. The historical field data used in this analysis are available as Supporting Information.

**Table 4:** Abbreviated data quality assessment (C = Cantarell, F = Forties, MS = Midway-Sunset, PB = Prudhoe Bay, W = Wilmington). * = Poor quality, ** = Moderate quality, *** = High quality.

	C	F	MS	PB	W
API Gravity	**	**	**	**	**
Associated Gas Processing Path	*	*	*	**	*
Fraction of remaining natural gas reinjected	***	NA	***	***	NA
Gas-Oil-Ratio	***	***	***	***	***
Heater/treater	*	*	*	**	*
Oil Production	***	***	***	***	***
Producing Wells	***	***	***	***	***
Reservoir Pressure	***	***	**	***	**
Stabilizer Column	*	*	*	**	*
Steam-Oil-Ratio	NA	NA	***	NA	***
Water Injecting Wells	NA	***	***	***	***
Water injection ratio	NA	***	***	***	***
Water-Oil-Ratio	***	***	***	***	***

**Table 5:** Abbreviated data sources (C = Cantarell, F = Forties, MS = Midway-Sunset, PB = Prudhoe Bay, W = Wilmington).

	C	F	MS	PB	W
API Gravity	[19]	[20]	[21]	[22]	[23]
Associated Gas Processing Path	-	-	-	[24]	-
Fraction of remaining natural gas reinjected	[18]	NA	[25]	[16, 26]	NA
Gas-Oil-Ratio	[18]	[27]	[25]	[16]	[28]
Heater/treater	-	-	-	[24]	-
Oil Production	[18]	[27]	[25]	[16]	[28]
Producing Wells	[18]	[29]	[25]	[16]	[28]
Reservoir Pressure	[18]	[30]	[31–33]	[34]	[35]
Stabilizer Column	-	-	-	[24]	-
Steam-Oil-Ratio	NA	NA	[25]	NA	[28]
Water Injecting Wells	NA	[36]	[25]	[26]	[28]
Water injection ratio	NA	[27, 36]	[25]	[16, 26]	[28]
Water-Oil-Ratio	[18]	[27, 37]	[25]	[16]	[28]

## Overview of data and modeling approaches for the fields

This section contains overviews of data acquisition, assumptions, and extrapolations for the five fields. For the calculation of transport-related energy investment flows it is assumed that Houston, U.S. is the destination for oil from all five fields. This assumption provides a constant basis of comparison, following Brandt et al. [8].

### Cantarell

**Fig 2:**
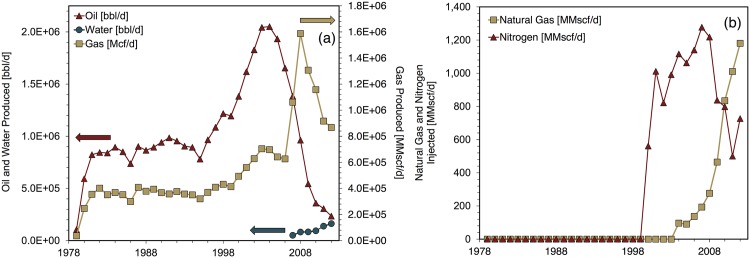
Cantarell input data for fluid production and injection. (a) Fluid production, (b) fluid injection. Source: [18].

A large portion of data regarding the Cantarell field was obtained from Lozada, Torres, et al. [18]. This source contains a graph (Fig 2 in the reference) with extensive historical data on Cantarell. The Cantarell modeling period is 1979-2012.

Gas lifting, nitrogen gas flooding, and natural gas reinjection are production methods used at Cantarell. Cantarell is the only field in this study in which nitrogen injection—which commenced in 2000 to maintain reservoir pressure [19]—is applied.

Modeling of gas lift at Cantarell (beginning in 1987 [38]) used data from Kettles, Kuo, et al. [39] that tracked the number of wells using gas lift from 1987 through 1995. During this period the proportion of total producers using gas lift increased steadily. As of 2001, Kettles et al. [39] indicate that all producers at Cantarell used gas lift. From 1987 through 1995 the proportion of wells on gas lift was calculated by first obtaining the total number of wells on gas lift from Kettles et al. [39]. The total number of all producers wells (naturally flowing and gas lifted) was obtained from Lozada et al. [18]. These figures were used to calculate the total proportion of wells on gas lift. Kettles et al. [39] also present data on total active producers, but their figures are slightly higher than the corresponding chart in Lozada et al. [18]. Using this method, 100% of Cantarell wells were on gas lift by 1995. It is assumed that during 1995-2012 100% of wells were on gas lift.

In 2001 an average of 2.8 MMscf/d was injected into each producer well for gas lift [39]; this value was used to compute the per-bbl lifting gas ratio, which was assumed to apply to all of the field’s gas lift wells.

Drilling activity for Cantarell was approximated by using Fig 2 from Lozada et al., which includes the number of production wells [18]. It is assumed that the number of wells drilled in a year is equal to the year-over-year increase in total wells. If the number of wells in Lozada et al. [18] declined year-over-year, then drilling activity for that year is set equal to zero. This method does not include drilling of injector wells (which are not listed in Lozada et al. [18]) and excludes some proportion of the wells that were drilled but inactive. This method will likely underestimate drilling activity at Cantarell.

Natural gas reinjection began at Cantarell in 2004 and injection volumes increase through the end of the modeling period [18]. Fig 2 from Lozada et al. includes data on natural gas production and reinjection [18]. In 2011 and 2012 Cantarell field data indicate that the proportions of gross natural gas reinjected are higher than the amounts of natural gas produced [18]. Because available technical literature did not indicate the importation of natural gas for flooding at Cantarell during these years, Cantarell is modeled using OPGEE’s iterative procedure to maximize FRNGI while maintaining zero gas imports. Fig 2 depicts key Cantarell field parameters during the the modeling period.

### Forties

The Forties field modeling period is 1975-1999. The U.K.’s Department of Energy & Climate Change (UK-DECC) was the source of most data for Forties [27, 29, 36, 37].

Production methods used in modeling the Forties field are water injection and water flooding, along with artificial lift via both downhole pumps and gas lift.

While water injection began in 1976 [36], no artificial lift methods were used for approximately the first thirteen years of production [40]. During 1989-1992, 47 wells were modified to accept gas lift [40, 41]. A new production platform was completed in 1987 and the following year its wells began using downhole pumps [40]. This information, along with the total number of active producer wells obtained from UK-DECC data [29], was used to estimate the proportion of naturally flowing wells, wells using downhole pumps, and wells using gas lift. If a Forties field well displayed any positive injection or production during a given year, it was assumed to be an “active” well. Well-level data indicated that active producers increased by nine from 1986-1988, reflecting the activation of the new production platform with downhole pumps.

During 1975-1987 no artificial lift methods are used at Forties. In 1988, submersible pumps are used in the newly activated producer wells. It is assumed that the proportion of non-pumped producer wells on gas lift increased linearly from 1989-1992. Beginning in 1992 all non-pumping producers use gas lift. The 1992 ratio of producer wells using downhole pumps to the ratio of producer wells using gas lift is also used to allocate producer wells from 1993-1999.

The gas lift injection ratio was estimated from a presentation by Apache Corporation, the current field operator, stating that a producer well received lifting gas at a rate of 1.4-2.6 million standard cubic feet per day (MMscf/d) [42]. An intermediate value of 2 MMscf/d was selected to compute per-bbl lifting gas injection.

**Fig 3:**
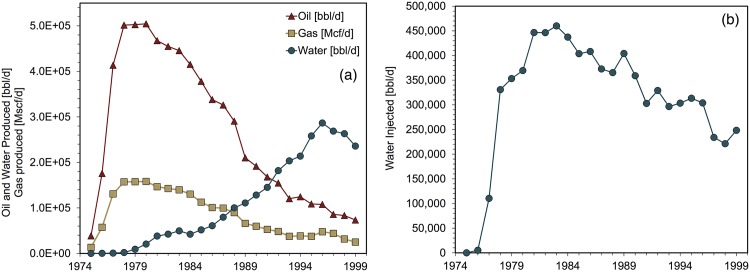
Forties input data for fluid production and injection. (a) Fluid production, (b) fluid injection Sources: [27, 36, 37].

The total number of wells drilled in Forties was available during 1975-1995 [40], as was the total number of wells drilled during 1992-2001 [43]. From this data the average number of wells drilled during 1996-2001 was calculated, and the number of wells drilled during 1997-1999 is set equal to this estimated figure. Fig 3 depicts key Forties field parameters during the the modeling period.

### Midway-sunset

Production data for the Midway-Sunset field were obtained from annual reports of California’s Division of Oil, Gas & Geothermal Resources (CA-DOGGR) [25]. CA-DOGGR annual reports began to include steam injection statistics in 1966. Because steam generation is expected to significantly impact energy consumption, the Midway-Sunset modeling period is 1966-2009.

Production practices included in the Midway-Sunset analysis include water reinjection, gas reinjection, and steam injection. Steam flooding and cyclic steam are distinct steam injection categories in CA-DOGGR records. Total steam injection was calculated by summing the steam flooding and cyclic steam figures. Use of downhole pumps is assumed for all producing wells due to Midway-Sunset’s low reservoir pressure and low overall API gravity of 19 degrees [21].

There were distinct phases of steam generation at Midway-Sunset. Produced crude oil served as the fuel for steam generation for more than two decades following the initiation of steam injection in 1960. Environmentally-driven legislation mandated the phase-out of crude oil-fired steam generation, leading to the use of natural gas for steam generation in California. This transition phase began in the early 1980s and eventually natural gas was burned to produce all steam [9].

After natural gas-fired steam generation began, some steam was generated using once-through steam generators (OTSG), while the remainder was generated using heat recovery steam generators (HRSG), which are installed in petroleum field cogeneration plants.

CA-DOGGR records contain complete cogeneration statistics starting in 1989 with capacities of plants reported back to 1983, when CA-DOGGR records indicate that the first cogeneration project became active [25]. The cogeneration projects produce from tens of thousands to one million lbs steam/hr. Data regarding the relative proportions of natural gas and crude oil used to generate total steam at Midway-Sunset was unavailable. It is assumed that all steam was generated using crude oil until the first natural gas cogeneration project began in 1983. Cogeneration capacity increased gradually from 1983-1988 and large capacity increases occurred in 1989 and 1990 [25].

The proportional increase in the total cogeneration capacity is used as a proxy to model the proportion of steam generated by burning natural gas. The cogeneration capacity in 1989 is used as a baseline; the percentage of this baseline capacity achieved during 1983-1988 is used as the overall proportion of steam generated by natural gas. It is assumed that all steam is generated using natural gas, either in an OTSG or through cogeneration in an HRSG, during 1989-2009.

**Fig 4:**
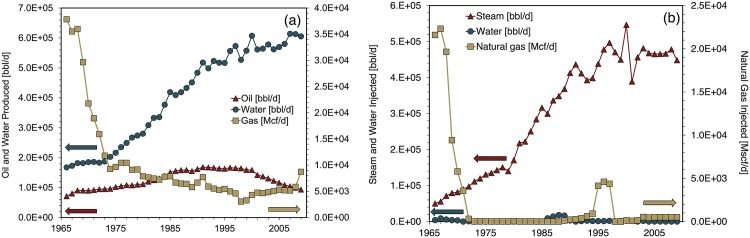
Midway-Sunset input data for fluid production and injection. (a) Fluid production, (b) fluid injection Source: [25].

Steam injection data for 1977 was not available from CA-DOGGR statistics [25]. 1977 steam injection is set equal to the average of steam injection in 1976 and 1978. Some natural gas reinjection was performed during the Midway-Sunset modeling period, however the associated energy requirements are orders of magnitude smaller than steam generation requirements and thus not modeled. Fig 4 depicts key Midway-Sunset field parameters during the modeling period.

### Prudhoe bay

Most data to model the Prudhoe Bay field were obtained from Alaska’s Oil and Gas Conservation Commission (AOGCC) [16, 22, 26, 44]. Prudhoe Bay’s modeling period is 1978-2004. Production methods used in Prudhoe Bay include water reinjection, water flooding, gas reinjection, and gas lifting. AOGCC data provided the proportion of active producing wells that used gas lift [44]. Reservoir pressure for Prudhoe Bay was obtained from a scatterplot in an article from the Office of the Federal Coordinator, Alaska Natural Gas Transportation Projects [34].

Gas reinjection occurs during every year of the model. The ratio of gas injected to gross gas produced is above 90% during every year [16, 26]. The lack of a natural gas pipeline connecting Alaska to external markets prompted the reinjection of a very high proportion of produced gas for enhanced oil recovery purposes [34]. The OPGEE iterative process is thus used to maximize FRNGI while avoiding gas imports during the entire Prudhoe Bay modeling period.

The Prudhoe Bay gas lift injection ratio is estimated using data from 12 gas lift wells in 1985, which indicated a lifting gas injection rate of 0.5-6 MMscf/d [45]. An intermediate value of 2 MMscf/d was chosen to estimate per-bbl lifting gas injection rates.

**Fig 5:**
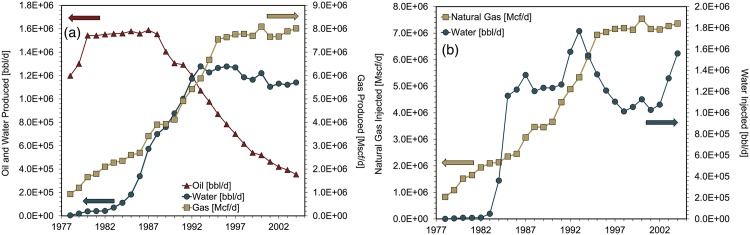
Prudhoe Bay input data for fluid production and injection. (a) Fluid production, (b) fluid injection Sources: [16, 26].

Drilling activity for Prudhoe Bay is approximated by analyzing the total “active completions” from AOGCC annual reports [44]. Active completions do not include injector wells. This method thus underestimates drilling energy requirements. Active completions were not available during 1985-1989; during these years active completions are estimated by linear interpolation of 1984 and 1990 values. The number of new wells drilled each year is assumed to be equal to the year-over-year increase in active completions. Fig 5 depicts key Prudhoe Bay field parameters during the modeling period.

### Wilmington

CA-DOGGR production data was obtained for California’s Wilmington field [28]. The Wilmington modeling period is 1956-2009. The entire Wilmington modeling period includes water injection, which began in 1953 [46]; steam injection has also occurred [28]. CA-DOGGR statistics also indicate small-scale reinjection of natural gas at Wilmington during 1956-1959 and 1994-1996 but the volumes injected are a small proportion (approximately 11% or less) of produced gas and therefore not included in the modeling process [28].

The literature indicates extensive use of downhole pumps at Wilmington dating back several decades. In 1937 pumps were used at 26% of the producers at Wilmington [47]. A 1945 analysis of producers that had undergone “multi-zone” completions stated that 42% used pumps; multi-zone completed wells constituted approximately 20% of the producers at that time [48]. An article about offshore operations at Wilmington in 1987 indicated that 100% of a company’s 906 producer wells used pumps [49]. CA-DOGGR records indicate that these 906 wells were 97% of active offshore producers in 1987 and 44% of all wells, onshore and offshore, at Wilmington [25]. Given the absence of more detailed data, it is assumed that all wells used downhole pumps during the entire Wilmington modeling period.

Some steam injection occurred in Wilmington (relatively minor in comparison to Midway-Sunset). Similarly to the method used for Midway-Sunset, it is assumed that crude oil was burned to generate all steam through 1982. The share of natural gas burned to generate steam is assumed to increase linearly from 1983-1988; from 1988-2009 all steam is generated using natural gas. CA-DOGGR statistics indicate only one cogeneration plant at Wilmington, operated from April 1989 to February 1999, and cogeneration was thus included in the model from 1989 through 1998 [28]. As with Midway-Sunset, steam injection values for 1977 are set equal to the average of the 1976 and 1978 values from CA-DOGGR statistics [28].

Detailed average reservoir pressure data over the modeling period was not available for Wilmington. However, Wilmington’s history allows for a reasonable estimate. Wilmington consists of both onshore and offshore portions in the Los Angeles-Long Beach, California area. Significant subsidence affected the urban developments above the reservoir within a decade of the commencement of production [35]. Operators began waterflooding operations by 1953, presumably for the specific purpose of increasing oil production rather than addressing subsidence *per se* [35].

Huey [35] presents data indicating the efficacy of the waterflooding program. Pressure values are given for most of the blocks comprising Wilmington at two different time periods. For each block the precise dates vary; the first date is during the 1958-1961 period and the second date is within the 1963-1964 period. Average reservoir pressures for most of the blocks within the Wilmington field indicate that waterflooding had increased reservoir pressures by the 1963-1964 period (though on average they were well below the original reservoir pressure values). It is assumed that waterflooding at Wilmington has maintained the reservoir pressures indicated by Huey [35] during the 1963-64 period.

**Fig 6:**
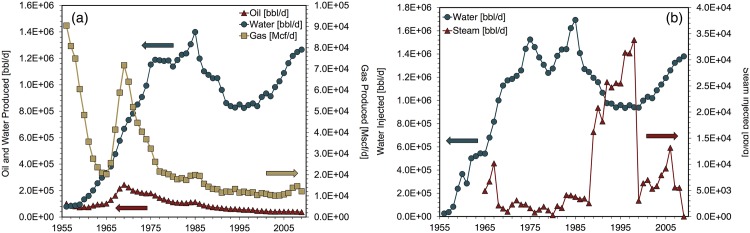
Wilmington input data for fluid production and injection. (a) Fluid production, (b) fluid injection Source: [28].

For modeling purposes the Wilmington reservoir pressure from 1956-1961 is set equal to the average of the reservoir pressure values given by Huey [35] during the 1958-1961 period. The model reservoir pressure from 1963-2009 is set equal to the average of Huey’s [35] reservoir pressure values during the 1963-1964 period. Reservoir pressure for 1962 is the average of the values for the 1958-61 period and the 1963-64 periods. Fig 6 depicts key Wilmington field parameters during the modeling period.

## Sensitivity analysis

Using OPGEE to estimate a field’s EROI is an approximate process involving the generalization of locally heterogeneous and uncertain reservoir parameters to field-level assessments. Furthermore, details regarding the surface processing of crude oil are not generally available.

As an example sensitivity analysis variable, consider OPGEE’s use of the productivity index, which directly affects energy requirements for downhole pumping [12]. OPGEE’s default injectivity ratio—which affects water injection energy requirements—is also set equal to the productivity ratio [12]. Downhole pumps and water injection are production practices modeled in Forties, Wilmington, Midway-Sunset, and Prudhoe Bay. For these fields sensitivity analysis was performed by using productivity ratios of 1 bbl/psi-d and 50 bbl/psi-d.

Sensitivity analysis was performed for each field for three time increments of the modeling period: early (the first three years), middle (the middle three or four years), and late (the final three years). The NER or EER for each time increment is calculated as the average of the values over each year of the increment.

Table 6 contains a summary of the sensitivity analysis settings.

**Table 6:** Summary of sensitivity analysis parameters (C = Cantarell, F = Forties, MS = Midway-Sunset, PB = Prudhoe Bay, W = Wilmington).

Parameter	Description	Fields	Notes
Gas Lift Injection Ratio	Varied +/- 25%. Base: Variable.	C, F, PB	
Processing Intensity	Crude Oil - High: Heater/treater and stabilizer column - Base: Stabilizer column - Low: None	C, F, MS, PB, W	a
	Natural gas - High/Base: Dehydrator, Acid Gas Removal Unit, and Demethanizer - Low: Dehydrator and Demethanizer		
Steam Gen. Efficiency	OTSG - High: 300°F outlet exhaust, 0.03 Btu/Btu fuel shell loss - Base: 350°F outlet exhaust, 0.04 Btu/Btu fuel shell loss - Low: 400°F outlet exhaust, 0.05 Btu/Btu fuel shell loss	MS, W	
	HRSG - High: 300°F outlet exhaust, 0.04 Btu/Btu fuel shell loss, turbine type D - Base: 350°F outlet exhaust, 0.05 Btu/Btu fuel shell loss, turbine type C - Low: 400°F outlet exhaust, 0.06 Btu/Btu fuel shell loss, turbine type B		
Well Diameter	2.50 inches and 3.05 inches. Base: 2.775 inches.	MS, W	
Productivity Index	1 bbl/psi-d and 50 bbl/psi-d. Base: 3 bbl/psi-d.	F, MS, PB, W	
Air Sep. Energy Intensity	Varied +/- 10%. Base: 0.0042 kWh/scf.	C	

^a^ For Prudhoe Bay processing sensitivity analysis is conducted with crude oil and associated gas processing varied independently. The low-intensity natural gas processing scheme for Prudhoe Bay consists only of the dehydrator.

## Results and discussion

All fields experienced substantial declines in estimated NER during their modeling periods. EER estimates were more varied. Four of the fields had moderate to severe EER declines. At Prudhoe Bay, the EER_*oil*_ declined only moderately but the EER_*total*_ increased over the modeling period. Within a field, the NER and EER ratios have very different profiles.

Production declines are an important cause of corresponding declines in the estimated EROI values. These declines affect the energy returns of the fields; the input charts in the Materials and methods section illustrate this fact for all five fields. This section analyzes the resulting EROI values in the context of both energy investment and energy returns trends for each field.

EER profile plots are available in Tripathi [1].

### Cantarell

**Fig 7:**
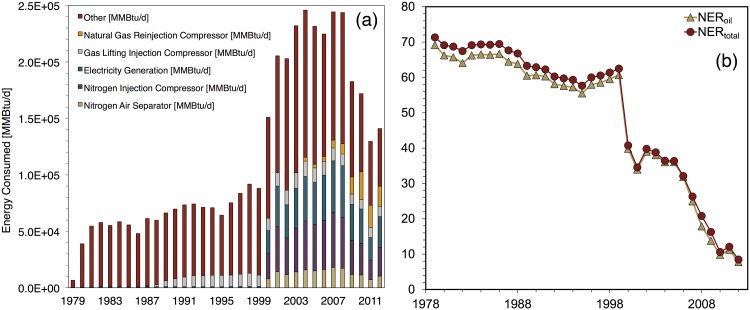
Cantarell base case results showing (a) total energy inputs for major processes (b) NER ratios.

Cantarell’s NER estimates (Fig 7) demonstrate two phases; the first spans 1979 to 1999. During these years, estimated NER values declined only by a moderate 12%. This phase included the transition of all producing wells from free-flow to gas lift.

The severe drop in estimated NER occurring in 2000 is the singular feature of Cantarell’s NER history. This decline coincides with the initiation of nitrogen injection. (Production of both oil and gas increased from 1999 to 2000 and thus production declines did not contribute to this significant one-year NER decline.) Fig 7 depicts the surge in energy investments flowing into Cantarell to enable nitrogen injection. Large new energy investment flows were required to operate the nitrogen air separator and nitrogen injection compressor. The amount of natural gas burned for electricity generation on-site grew as well. The sharp decline in oil production from 2004 onward (see Fig 2) drove the following NER decline. Reinjection of natural gas began at Cantarell during 2004. In the final years of the model run the natural gas reinjection compressor becomes a significant contributor total energy consumption as well.

In the OPGEE model, the energy used to construct petroleum field materials (embodied energy) and the energy used to transport crude oil for refining are both directly proportional to the volume of crude oil produced [12]. In the case of offshore fields such as Cantarell, it is assumed that most petroleum field operations are powered using produced natural gas. Therefore, embodied energy, energy to drill wells, and energy to transport produced crude oil are the only sources of external energy costs at Cantarell. Thus, as the energy content of Cantarell’s produced crude oil declines, there is a similarly proportional drop in external energy investments that flow into the system to ship the produced crude oil; there is also a proportional decrease in embodied energy costs. This behavior causes Cantarell’s EER to remain relatively stable (see [1]). Fluctuations in annual drilling activity contribute to minor variation occurring in most of the modeling period.

EER_*total*_ rises in 2007 and 2008 due to increasing natural gas production. Natural gas production in 2008 was more than twice as large as in 2006. Furthermore, a significant proportion of this natural gas remained after processing and was exported. Natural gas is reinjected in the most recent years, reducing both NER and EER.

### Forties

**Fig 8:**
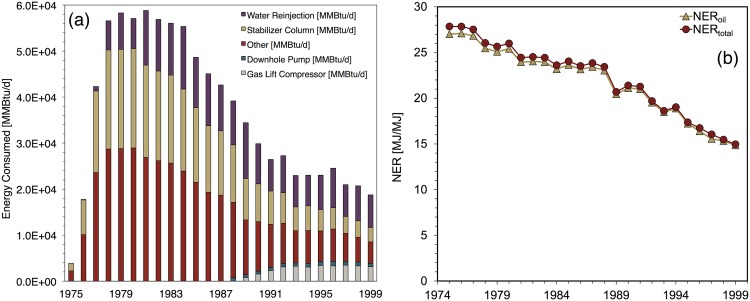
Forties base case results showing (a) total energy inputs for major processes (b) NER ratios.

Estimated NER values for Forties (Fig 8) declined over the 25-year modeling period. NER_*total*_ declined by 46% and NER_*oil*_ fell by 45%. There are two general phases in the Forties NER estimates. The first phase is from 1974-1988; the NER_*total*_ declined at an average annual rate of 1.1% in this period. From 1988-1999 the NER_*total*_ average annual decline rate was 3.0%. The transition corresponds to the period during which both downhole pumping (in 1988) and gas lift (1989-1992) began. Fig 8 displays the rise in energy usage for the gas lifting compressor and downhole pumps during this transition. The energy costs of these artificial lifting technologies were significant but relatively small compared to the costs of nitrogen injection at Cantarell.

EER_*total*_ values during Forties’ early and middle periods are relatively insensitive to parameter variation. During the late period, however, all parameters noticeably affect EER_*total*_ values, because during this period Forties became a natural gas importer in the base case scenario. The magnitude of imports was very low in the final two years of the modeling period, thus small changes in the onset and magnitude of natural gas imports can cause large variation in EER_*total*_ values. See [1] for graphical EER results for Forties.

### Midway-sunset

**Fig 9:**
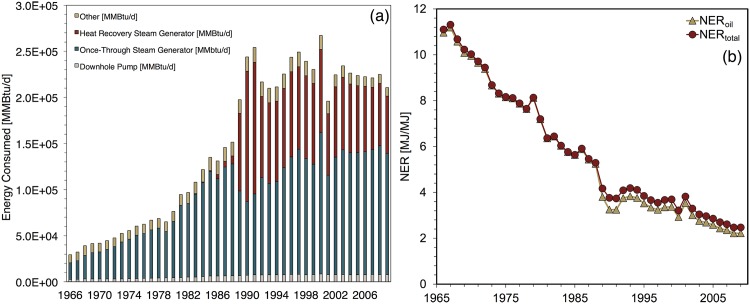
Midway-Sunset base case results showing (a) total energy inputs for major processes (b) NER ratios.

Fig 9 contains estimated NER values for Midway-Sunset. From 1966-2009 estimated NER_*total*_ values declined relatively steadily, from 11.8 to 2.5 at an annual average rate of 1.8%. NER_*oil*_ values fell similarly. The largest year-over-decline in NER_*total*_—21%—occurred between 1988 and 1989. This decrease coincides with the largest single-year increase in cogeneration capacity. Because rising cogeneration capacity is used as a proxy for the rising share of steam generated by natural gas, the share of natural gas-fired steam also increased significantly between 1988 and 1989 from 68% to 100%.

Midway-Sunset’s EER values were steady during 1966-1972 (see [1]). During 1972-1989 EER values declined significantly. This period included Midway-Sunset’s transition to a natural gas importer in 1973. Natural gas imports continued to rise during this period, mostly due to the rising proportion of steam generated by natural gas. The most abrupt EER decline occurred 1988-1989, caused by the large increase in the share of natural gas-generated steam occurring in that year. From 1989-2006, the EER values decline gradually.

Fig 9 depicts Midway-Sunset’s energy consumption profile over the modeling period. Energy for steam generation (HRSG and OTSG) dominates energy consumption during the entire modeling period. The rise in energy consumed for steam generation parallels the rise in the Midway-Sunset steam-oil-ratio from less than 1 bbl steam per bbl oil to nearly 5 bbl/bbl over the modeling period. Notably, downhole pumping at Midway-Sunset composed less than 5% of total energy consumption during 1983-2009.

### Prudhoe bay

**Fig 10:**
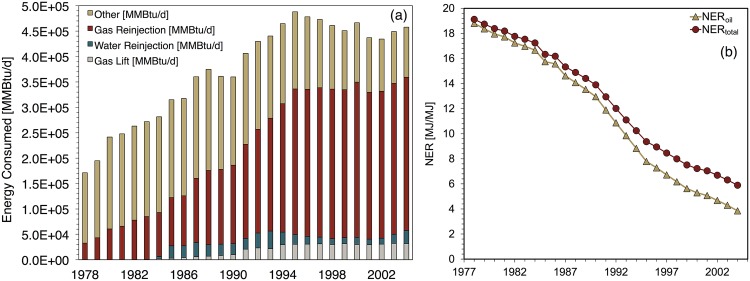
Prudhoe Bay base case results showing (a) total energy inputs for major processes (b) NER ratios.

Prudhoe Bay’s estimated NER_*total*_ (Fig 10) declined by 69% over the modeling period from its initial value of 19.1; its estimated NER_*oil*_ declined by 80% from its initial value of 18.8. Notably, Prudhoe Bay’s NER_*total*_ and NER_*oil*_ values were nearly identical at the beginning of the modeling period but they diverge due to the higher decline rate of the NER_*oil*_ values. This behavior results from the evolving production profile of Prudhoe Bay.

The Prudhoe Bay gas-oil-ratio increased from 778 scf/bbl oil to 22,614 scf/bbl during the modeling period—a 29-fold increase. The base case scenario for Prudhoe Bay assumes use of the demethanizer, which extracts natural gas liquids (NGL) from the produced gas. In contrast to natural gas, which is reinjected, NGLs at Prudhoe Bay are modeled as being combined with crude oil and exported. Rising NGL production and exports over the modeling period thus contribute to a more gradual decline in NER_*total*_.

Fig 10 depicts energy consumption at Prudhoe Bay. An important feature in this figure is the gradual rise of the energy consumption share of the natural gas reinjection compressor, which grew from 15% to 66% during 1978-2004. Prudhoe Bay’s high natural gas production and the ultimate reinjection of most of this produced gas serve to make gas reinjection the dominant energy consumption source for most of the modeling period. This feature is unique to Prudhoe Bay among the five fields considered in this study.

Prudhoe Bay’s estimated EER_*oil*_ profile is relatively unchanged over the modeling period. As with Cantarell and Forties, Prudhoe Bay receives relatively few external energy investments. An unusual feature of Prudhoe Bay is the 44% rise in its estimated EER_*total*_ over the modeling period (see [1]). This behavior is caused by Prudhoe Bay’s rising NGL exports resulting from its increasing natural gas production. While total energy output at Prudhoe Bay declined by 55% over the modeling period, total external energy investments and associated fuel cycle investments declined by 68%. Declining crude oil production resulted in approximately proportional total declines in external energy investments required for transportation and embodied energy. Note that OPGEE does not track volumes of NGLs when it calculates transport-related energy costs [12].

### Wilmington

**Fig 11:**
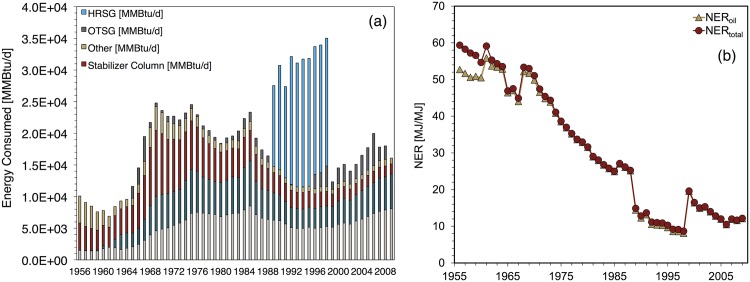
Wilmington base case results showing (a) total energy inputs for major processes (b) NER ratios.

Fig 11 shows NER estimates for Wilmington. NER_*total*_ declined from 59.4 to 12.2 during this period; EER_*total*_ fell from 508 to 22.9. The early years of the modeling period (1955-1964) depict the onset of the waterflooding projects described above.

During 1955-1964 NER_*total*_ values are fairly steady. The moderately lower NER_*total*_ values in 1965-1967 are due to increases in steam injection volume during those years. NER_*total*_ values decline fairly steadily from 1968-1988 and then exhibit sharply lower values during 1989-1998. The 1989-1998 period corresponds to a period of significantly higher steam injection volumes and the operation of a cogeneration plant for steam generation discussed above. The termination of the cogeneration plant in 1999 coincided with a significant reduction in overall steam injection and an increase in NER_*total*_. From 1999 onward NER_*total*_ declines gradually.

A sharp decline in EER values occurs from 1973 to 1977 (see [1]). In this period EER_*total*_ falls from 375 to 80, caused by Wilmington’s shift from natural gas exports to natural gas imports. This shift was caused by a 47% decline in Wilmington’s natural gas production from 1973 to 1977. Increases to Wilmington’s energy consumption did not cause it to become a natural gas importer; its total consumption was actually 4% lower in 1977 than in 1973.

Fig 11 also depicts energy consumption at Wilmington. In the first year of the modeling period, 1956, the crude oil stabilizer column was the single largest consumer of energy. In the following years the rate of water injection and water pumped increased significantly. By 2009, the final year of the modeling period, downhole pumping and water injection pumping accounted for 84% of total energy consumption at Wilmington. Steam generation’s dominant role during the 1989-1998 period is evident in Fig 11.

### Sensitivity analysis results

Table 7 depicts sensitivity analysis impacts on estimated NER_*total*_ for each of the five fields. As noted above, the early, middle and late periods represent the first three years, middle three (or four) years, and last three years of each modeling period. Columns “Early Low” through “Late High” contain the NER_*total*_ resulting from parameter variation divided by the time period’s base case NER_*total*_, in percentage format. Note that NER_*total*_ is unchanged for several field/time period/sensitivity parameter combinations, resulting in 100% values in Table 7. For example, in the early Cantarell modeling period varying the nitrogen separator’s energy usage rate does not impact NER_*total*_ because nitrogen injection had not yet begun.

**Table 7:** Sensitivity analysis results for NER_*total*_ at each field and time period.

Field	Parameter	Range Low	Range High	Early Low	Early High	Middle Low	Middle High	Late Low	Late High
Cantarell	Gas Lift Injection Ratio	-25%	+25%	100%	100%	96%	104%	99%	102%
Cantarell	Nitrogen Separation Power Rate	-10%	+10%	100%	100%	100%	100%	97%	103%
Cantarell	Oil & Gas Processing Intensity	High	Low	81%	215%	84%	191%	97%	103%
Forties	Gas Lift Injection Ratio	-25%	+25%	100%	100%	100%	100%	96%	104%
Forties	Productivity Index	1	50	100%	100%	100%	116%	92%	135%
Forties	Oil & Gas Processing Intensity	High	Low	91%	130%	92%	125%	94%	120%
Midway-Sunset	Steam Generation Efficiency	High	Low	99%	101%	97%	103%	96%	105%
Midway-Sunset	Productivity Index	1	50	100%	100%	100%	100%	100%	100%
Midway-Sunset	Oil & Gas Processing Intensity	High	Low	96%	110%	98%	104%	99%	102%
Midway-Sunset	Well Diameter	3.05	2.5	100%	100%	100%	100%	100%	100%
Prudhoe Bay	Gas Lift Injection Ratio	-25%	+25%	100%	100%	99%	101%	99%	101%
Prudhoe Bay	Productivity Index	1	50	100%	100%	95%	103%	94%	104%
Prudhoe Bay	Crude Oil Processing Intensity	High	Low	88%	106%	92%	104%	97%	101%
Prudhoe Bay	Gas Processing Intensity	High	Low	84%	99%	66%	95%	49%	72%
Wilmington	Productivity Index	1	50	96%	101%	63%	138%	58%	152%
Wilmington	Oil & Gas Processing Intensity	High	Low	85%	183%	91%	131%	96%	112%
Wilmington	Well Diameter	3.05	2.5	100%	100%	100%	100%	100%	100%
Wilmington	Steam Generation Efficiency	High	Low	100%	100%	100%	100%	100%	100%

Variation of the oil and gas processing scheme had the greatest impact on Cantarell’s NER_*total*_ in the early and middle modeling periods. Parameter variation had insignificant impact on Cantarell’s NER_*total*_ in the late modeling period.

During all three time periods at Forties, the low-intensity oil and gas processing case resulted in significantly higher NER_*total*_ values because of energy savings resulting from turning off the stabilizer column. In the middle and late modeling periods, raising the productivity index from the default of 3 bbl-psi/d to 50 bbl-psi/d significantly raised the NER_*total*_. The volume of water injected into the Forties field was significantly higher in the middle and late modeling periods. During these periods reservoir pressure had also declined below early period levels. These factors lead to less energy consumption for water reinjection in the 50 bbl/psi-d case.

At Midway-Sunset, variation of oil and gas processing caused moderate changes to NER_*total*_ values in the early modeling period. NER_*total*_ was not very sensitive to parameter changes in the middle and late modeling periods. Giving the primary role of steam generation in energy consumption at Midway-Sunset, the relative insensitivity to steam generation is notable. Wider variation of steam generation efficiency parameters would have led to greater changes in NER_*total*_ and EER_*total*_ values, although these ranges were selected to simulate reasonable physical bounds on steam generator engineering (i.e., it does not make sense to assume a steam generator 25% less efficient than the default, as such a steam generator would likely not be built).

For Prudhoe Bay, variation of the natural gas processing scheme significantly impacts NER_*total*_ at all modeling periods. Uniquely among the five fields, Prudhoe’s Bay’s default gas processing scheme does not include use of the acid gas removal unit (AGR). In the high-intensity gas processing scheme the AGR unit is turned on and becomes a large source of energy consumption. This behavior results both from the high volume and significant acid gas content of its produced gas stream. The OPGEE AGR unit’s energy consumption is directly proportional to the acid gas—either H_2_S or CO_2_—content of the input stream [14]. While the gas composition used to model Prudhoe Bay does not contain H_2_S, it contains 12% CO_2_.

For the case of Wilmington, the oil and gas processing intensity had a significant impact on NER_*total*_ in the early and middle modeling periods, mostly from the effect of turning off the stabilizer column.

## Conclusion

All five fields analyzed in this study exhibit significant declines in NER/EROI over time. The temporal declines in EROI estimates observed in this study result both from decreasing oil and gas production and increasing energy investments required for processing and handling fluids.

NER values declined significantly for all fields. EER estimates were more complex. NER and EER estimates have notable differences: NER values include internal energy flows and thus more completely capture the energy requirements of secondary and tertiary production methods. Compared to NER values, EER values are subject to much larger uncertainty stemming from small modeling changes.

Poor data availability regarding the oil and gas processing scheme was a significant source of uncertainty regarding NER estimates, particularly earlier in the modeling periods. Improved estimates of reservoir pressure would reduce modeling uncertainty, especially in the case of Wilmington where water injection is a major energetic cost.

In the future OPGEE may be improved by considering NGL exports when calculating the energetic cost of petroleum transport. OPGEE’s calculation of the embodied energetic cost of injection wells in cases without water injection could also be improved.

The results of this analysis suggest further opportunities for temporal EROI estimates of oil and gas fields. Other fields with similar reservoir properties and production processes may be analyzed. For example, Midway-Sunset is a heavy oil field for which steam generation is the primary energy investment. How does its temporal EROI profile compare to those of similar fields? Kern River and South Belridge are two other large California fields with heavy oil and extensive histories of steam injection. CA-DOGGR records are comprehensive and readily allow for expansion of this analysis to these additional fields. Further analysis of steam injection-dominated fields may allow for the ultimate generation of “EROI temporal type curves” for particular combinations of reservoirs and production parameters, such as “heavy oil/steam injection.”

Also, as discussed above, many temporal EROI analyses use an economic approach of applying energy intensity factors to financial expenditures. The Daqing field study by Hu et al. is an example [7]. Using OPGEE to estimate Daqing’s EROI could allow for a preliminary comparison of the two approaches.

Importantly, OPGEE’s calculations of the indirect energy costs embodied in the construction of petroleum field materials should be expanded to include additional categories such as drilling machinery.

## Supporting information

S1 File Supporting information file S1_File.xlsx contains additional input data and results in numerical form. (XLSX)
